# Correction: Fabrication and behaviors of CdS on Bi_2_MoO_6_ thin film photoanodes

**DOI:** 10.1039/d2ra90113g

**Published:** 2022-11-16

**Authors:** Hao Yang, Zhiliang Jin, Hongyan Hu, Gongxuan Lu, Yingpu Bi

**Affiliations:** School of Chemistry and Chemical Engineering, Beifang University of Nationalities Yinchuan 750021 P. R. China; State Key Laboratory for Oxo Synthesis and Selective Oxidation, Lanzhou Institute of Chemical Physics, Chinese Academy of Science Lanzhou 730000 P. R. China

## Abstract

Correction for ‘Fabrication and behaviors of CdS on Bi_2_MoO_6_ thin film photoanodes’ by Hao Yang *et al.*, *RSC Adv.*, 2017, **7**, 10774–10781, https://doi.org/10.1039/C6RA28323C.

The authors regret that an incorrect grant number was shown in the acknowledgements section of the published article. The corrected section should read:

This work was financially supported by the Chinese National Natural Science Foundation (21603247, 41663012, 21263001 and 21463001).

The Royal Society of Chemistry apologises for these errors and any consequent inconvenience to authors and readers.

## Supplementary Material

